# Analysis of the TIL gene family in Brassicaceae species and functional study of *BrTIL1* in cold tolerance

**DOI:** 10.3389/fpls.2026.1794987

**Published:** 2026-02-27

**Authors:** Zhengnan Xu, Xiaolei Tao, Yanxia Xu, Abbas Muhammad Fahim, Yifan Wang, Hao Sun, Shiyi Li, Yuanyuan Zhang, Lijun Liu, Junyan Wu, Wancang Sun, Li Ma

**Affiliations:** 1 State Key Laboratory of Aridland Crop Science/College of Agronomy, Gansu Agricultural University, Lanzhou, China; 2 College of Life Sciences, Sichuan University, Chengdu, China

**Keywords:** abiotic stress, BrTIL1, cold stress, functional analysis, TIL gene family

## Abstract

Temperature-induced lipocalins (TILs) are a class of thermoregulated lipid-transporting proteins crucial for plant stress responses. However, systematic research on the TIL gene family remains relatively limited. In the present study, we conducted a comparative analysis of the TIL gene family in five Brassicaceae species (Arabidopsis thaliana, Brassica rapa L., Brassica rapa subsp. pekinensis, Brassica juncea L., and Brassica napus L.), identifying a total of 23 TIL genes. Analyses of their gene structures, evolutionary relationships, conserved motifs, and cis-acting elements showed extensive collinearity, close homology, and functional conservation, implying they may possess similar biological functions across different Brassicaceae species. The Brassica rapa TIL1 (BrTIL1) gene was significantly upregulated under low-temperature stress. Functional validation showed that Arabidopsis thaliana plants overexpressing BrTIL1 exhibited higher survival rates, soluble protein levels, and peroxidase (POD), catalase (CAT), and superoxide dismutase (SOD) activities under low-temperature conditions, confirming that BrTIL1 positively regulates cold tolerance. The BrTIL1 protein was localized to the cell membrane. A yeast two-hybrid screen identified six proteins interacting with BrTIL1. The genes encoding these interacting proteins exhibited differential expression under low-temperature stress, suggesting they may affect the functional activity of BrTIL1. In summary, this study provides a systematic analysis of the TIL gene family in five Brassicaceae species, elucidates the role of BrTIL1 in cold tolerance, and establishes a foundation for deciphering the molecular mechanisms of the cold stress response in Brassicaceae species.

## 1 Introduction

Temperature-induced lipocalins (TILs) constitute an ancient protein family with diverse functions (Åkerstrom et al., 2000). The characteristic structure of this family features three conserved SCR (Structurally Conserved Region) domains and eight antiparallel *β*-sheets, with two conserved glycosylation sites at the N-terminus (Charron et al., 2005). First identified in *Arabidopsis thaliana* as responsive factors to temperature changes, TILs were subsequently discovered in other plant species. Their general molecular function involves binding and transporting hydrophobic ligands (Åkerstrom et al., 2000), thereby participating in plant growth and development (Dickinson et al., 2021), and playing a crucial role in modulating plant responses to abiotic stresses (Boca et al., 2014). In the model plant *Arabidopsis thaliana*, the *AtTIL1* gene is induced by cold and salt stresses (Frenette Charron et al., 2002; Abo-Ogiala et al., 2014), and its homologous genes are similarly expressed during cold acclimation in major crops such as wheat (*Triticum aestivum* L.) and rice (*Oryza sativa* L.) (Frenette Charron et al., 2002; Ji et al., 2024).

As a lipid transport protein, the TIL protein has been demonstrated to improve abiotic stress tolerance in *Arabidopsis thaliana* and rice by protecting cell membranes from reactive oxygen species (ROS) damage and maintaining lipid homeostasis (Abo-Ogiala et al., 2014; Ji et al., 2024). Consistently, studies in tomato (*Solanum lycopersicum* L.), eucommia (*Eucommia ulmoides Oliver*), and alfalfa (*Medicago sativa* L.) have confirmed that TIL genes regulate physiological responses such as alterations in stress-responsive enzyme activities and reduced malondialdehyde (MDA) content, thereby enhancing plant tolerance to abiotic stresses (He et al., 2015; Zheng and Liu, 2022; Wu and Zhao, 2023). Despite these advances, reports regarding the molecular regulatory mechanisms of TIL genes are still limited. In chrysanthemum, *DgTIL1* undergoes post-translational modification (PTM) under cold stress, which enhances its interaction with DgnsLTP, stabilizes DgnsLTP protein, and ultimately modulates the expression and activity of peroxidase (POD). This regulatory cascade further improves cold tolerance in chrysanthemum (Huang et al., 2021). Nevertheless, systematic analyses of the TIL gene family are still scarce in Brassicaceae species (Frenette Charron et al., 2002; Charron et al., 2005). Furthermore, the specific functional role of TIL genes in regulating cold tolerance in rapeseed remains unclear. These knowledge gaps hinder our ability to fully exploit TIL genes in breeding cold-tolerant rapeseed varieties.

Rapeseed, as the world’s second-largest oilseed crop, plays a vital role in supplying edible vegetable oil and feed protein (Zheng and Liu, 2022). However, in northern China, winter temperatures can plummet to -32 °C, making low-temperature stress a primary limiting factor for rapeseed yield and severely threatening regional agricultural productivity (Tao et al., 2023). Additionally, rapeseed is widely utilized as a vital winter cover crop. Ecologically, winter rapeseed cultivation effectively increases vegetation cover during winter and spring, improves land cropping intensity and utilization efficiency, while significantly mitigating soil wind erosion exacerbated by bare winter soil (Liu et al., 2022). Economically, developing the winter rapeseed industry revitalizes idle winter agricultural resources, promotes balanced local economic development, and ultimately achieves a win-win outcome for ecological conservation and economic benefits (Zheng et al., 2022). Cold stress is a major environmental constraint limiting the growth, development, and geographical distribution of rapeseed in northern China (Zhao et al., 2023; Chen et al., 2025). When rapeseed is subjected to cold stress, cold signals are detected by membrane-localized sensors. Cold stress signal transduction then activates downstream signaling cascades, subsequently triggering key transcription factors that regulate the expression of cold-responsive genes (Song et al., 2024; Wang et al., 2024; Zhou et al., 2025). Concomitantly, a series of physiological changes are induced, including alterations in membrane lipid fluidity and reactive oxygen species (ROS) bursts, which further lead to membrane lipid peroxidation and elevated malondialdehyde (MDA) levels (Morales and Munné-Bosch, 2019). These physiological perturbations reduce photosynthetic rates, disrupt photosynthetic electron transport, and result in the accumulation of superoxide anion radicals (O^2−^) and hydrogen peroxide (H_2_O_2_) (Bai et al., 2022). Additionally, cold stress induces increases in soluble protein and sugar contents, changes in the activities of stress-responsive enzymes (Şahin-Çevik and Moore, 2012), modifications in endogenous hormone levels, and enhanced expression of cold-tolerance-related genes (Soorni et al., 2022). Therefore, elucidating the molecular mechanisms underlying rapeseed cold tolerance is of great scientific significance and urgent agricultural value for breeding stress-resistant rapeseed varieties.

Although TIL genes are crucial for plants’ response to abiotic stress, a comprehensive and comparative analysis of this gene family within the Brassicaceae is lacking. To address this gap, we performed a genome-wide identification and bioinformatic analysis of TIL genes across five Brassicaceae species. Using transcriptome data and RT-qPCR, we identified a key low-temperature-responsive gene *BrTIL1*, and validated its role in cold tolerance through functional assays. Furthermore, we constructed a yeast two-hybrid cDNA library to identify proteins that interact with *BrTIL1* and used RT-qPCR to analyze the expression patterns of the corresponding genes. Collectively, our findings elucidate the evolutionary mechanisms of the TIL gene family in Brassicaceae and provide valuable genetic resources for the molecular breeding of stress-tolerant crops. This study also establishes a foundation for deciphering the molecular regulatory network governed by *BrTIL1* in response to environmental stress.

## 2 Materials and methods

### 2.1 Plant materials and treatments

Two *Brassica rapa* cultivars with contrasting cold tolerance, the cold-tolerant ‘Longyou 7’ and the cold-sensitive ‘Lenox’, were used as experimental materials in this study (Xu et al., 2024; Fahim et al., 2025). Following germination, seedlings were transplanted into nursery pots and grown in a growth chamber under a 14-hour light (25 °C)/10-hour dark (20 °C) cycle until they reached the seventh leaf stage. Plants were then subjected to a low-temperature treatment of 0 °C for 6, 12, and 24 hours, with three biological replicates per treatment time point. Leaf samples were collected from both control (ambient temperature) and treated plants for subsequent RNA extraction and reverse transcription-quantitative PCR (RT-qPCR) analysis.

### 2.2 Genome-wide identification and physicochemical properties of TIL genes

The genome and annotation files for *Arabidopsis thaliana*, *Brassica rapa* subsp. *pekinensis*, *Brassica juncea*, and *Brassica napus* were retrieved from the Ensembl Plants (http://plants.ensembl.org/index.html) database (Bolser et al., 2016). Genomic data for *Brassica rapa* were provided by our research group. TIL proteins from these five species were identified using HMMER v3.0 with the TIL domain (PF08212) Hidden Markov Model (HMM) profile from the PFAM (http://pfam.xfam.org/) database (Mistry et al., 2021; Yang et al., 2024), using a default E-value threshold. The physicochemical properties of the identified TIL proteins were analyzed using the ExPASy ProtParam tool (https://web.expasy.org/protparam/) (Zhang et al., 2025), and their subcellular localizations were predicted using the WoLF PSORT (https://psort.hgc.jp/) online tool (Liu et al., 2023).

### 2.3 Bioinformatics analysis of the TIL gene family

A phylogenetic tree of the identified TIL genes was constructed using MEGA11 software and visualized using the iTOL (Letunic and Bork, 2021; Tamura et al., 2021). The protein motifs and conserved domains of the TIL proteins were analyzed using MEME software and the NCBI’s Batch CD-Search tool (http://www.ncbi.nlm.nih.gov/Structure/bwrpsb/bwrpsb.cgi), respectively. The cis-acting elements in the promoter regions of the TIL genes were identified using the Plant CARE (http://bioinformatics.psb.ugent.be/webtools/plantcare/html/) database (Chen et al., 2023). The chromosomal locations of the TIL genes were mapped using TB tools software. Tandem and segmental duplication events were investigated using the MCScanX module in TB tools with default parameters (Wang et al., 2013). The non-synonymous to synonymous substitution rate ratios (Ka/Ks) for the duplicated gene pairs were calculated using the Ka/Ks Calculator tool in TB tools (Chen et al., 2020). Finally, interspecies synteny relationships were analyzed using the Amazing Super Circos tool in TBtools-II. All visualizations were generated using TBtools-II software.

### 2.4 Analysis of TIL gene expression patterns

RNA-seq expression data for five tissues (root, stem, leaf, flower, and silique) of *Brassica napus*, *Brassica rapa*, and *Brassica juncea* were obtained from the BnIR (https://yanglab.hzau.edu.cn/), BRAD (http://brassicadb.cn/#/), and BjuIR (https://yanglab.hzau.edu.cn/BjuIR) databases, respectively, and subsequently analyzed (Chen et al., 2022; Yang et al., 2023; Zhang et al., 2024). For low-temperature stress expression analysis, transcriptome data (generated in our laboratory) from *Brassica rapa* and *Brassica napus* were used. All results were visualized using TBtools-II software (Chen et al., 2020).

### 2.5 RT-qPCR analysis

Total RNA was extracted using an RNA Pure Plant Kit (Tiangen, Beijing, China). Complementary DNA (cDNA) was synthesized from the extracted RNA using a reverse transcription kit (Tiangen). Quantitative real-time PCR was performed using the FastReal qPCR PreMix (SYBR Green) kit (Tiangen, Beijing, China) according to the manufacturer’s instructions. Actin (gene ID: 103850356) was used as the reference gene for normalization, and all primers used are listed in **Supplementary Table 1** (Tao et al., 2023). Relative gene expression levels were calculated using the 2^−ΔΔCT^ method.

### 2.6 Prediction and analysis of the *BrTIL1* protein-protein interaction network

The BrTIL1 protein sequence was submitted to the STRING database (http://string-db.org) (Szklarczyk et al., 2023) for protein-protein interaction (PPI) network, using *Brassica rapa* as the reference model with a Confidence threshold of 0.4. The resulting network was visualized and refined using Cytoscape software (v3.10.3), wherein isolated nodes (those without any interactions) were removed (Ono et al., 2025).

### 2.7 Subcellular localization of *BrTIL*

The coding sequence (CDS) of *BrTIL1* was cloned and ligated into the pSuper1300-GFP vector to generate a pSuper1300-*BrTIL1*-GFP fusion construct. This construct, along with a plasma membrane marker and the empty pSuper1300-GFP vector as controls, was transiently expressed in tobacco (*Nicotiana benthamiana*) leaves via *Agrobacterium tumefaciens*-mediated transformation. After 2–3 days, GFP fluorescence was observed using confocal laser scanning microscopy. To confirm membrane localization, the leaf tissues were subsequently treated with a 50% sucrose solution for 20 minutes to induce plasmolysis and then re-examined under the confocal microscope.

### 2.8 Preliminary functional validation of *BrTIL1*

The full-length cDNA of *BrTIL1* was amplified by PCR and cloned into the pDONR vector using a BP recombination reaction. The entry clone was then transferred into the pEarlyGate101 destination vector via an LR recombination reaction to generate an expression construct for plant transformation. The resulting plasmid was introduced into *Agrobacterium tumefaciens* strain GV3101 for floral dip transformation of *Arabidopsis thaliana*. Homozygous T3 generation plants were selected using 0.01% Basta (Marques and Gallazzini, 2024). These transgenic plants, along with wild-type controls, were subjected to cold treatment at -4 °C for 0, 3, 6, 12, and 24 hours. Following treatment, a subset of plants was used for RNA extraction and measurement of physiological parameters, including soluble protein (SP) concentration and antioxidant enzyme activities (SOD, POD, CAT). Another subset was returned to normal growth conditions for a one-week recovery period before phenotypic observation and survival rate analysis were conducted. The experiment included three biological replicates.

### 2.9 Yeast two-hybrid assay

To validate protein-protein interactions for *BrTIL1* in yeast, the yeast two-hybrid assay was performed. The constructed plasmids include the positive control (pGBKT7-53 + pGADT7-T), negative control (pGBKT7-Lam + pGADT7-T), and the experimental group (pGBKT7-BrTIL1 + pGADT7) were individually transformed into the *Saccharomyces cerevisiae* Y2HGold strain. The transformed yeast cells were plated on double-dropout (DDO) medium (SD/-Leu/-Trp) containing X-α-Gal and incubated at 30 °C for 3–5 days. Subsequently, single colonies were picked and spotted onto quadruple-dropout (QDO) medium (SD/-Ade/-His/-Leu/-Trp) supplemented with X-α-Gal and Aureobasidin A. After incubation at 30 °C for 3–5 days, the results indicated that the pGBKT7-BrTIL1 bait construct did not exhibited self-activation. Positive blue colonies from the screening were selected for one-to-one interaction validation.

## 3 Results

### 3.1 Identification and analysis of the TIL gene family

A total of 23 TIL genes were identified across the five Brassicaceae species: one in *Arabidopsis thaliana* (*AtTIL*), three in *Brassica rapa* (*BrTIL1–3*), five in *Brassica rapa* subsp. *pekinensis* (*BraTIL1–5*), six in *Brassica juncea* (*BjTIL1–6*), and eight in *Brassica napus* (*BnTIL1–8*). The genes were named systematically according to their chromosomal locations. Physicochemical analysis showed that these TIL proteins ranged from 137 (*BraTIL5*) to 346 amino acids (*BraTIL4*, *BnTIL4*, *BjTIL3*). Their molecular weights ranged from 15.7 kDa (*BraTIL5*) to 39.0 kDa (*BnTIL4*). All proteins exhibited an instability index greater than 31.72, and their theoretical pI values ranged from 4.9 (*BnTIL1*) to 9.25 (*BraTIL5*). Subcellular localization predictions indicated that the TIL proteins from *Arabidopsis thaliana* and *Brassica rapa* were localized to the cytoplasm. For the other species, cytoplasmic localization was predicted for three (60%) *BraTIL* proteins, four (66.7%) *BjTIL* proteins, and six (75%) *BnTIL* proteins (**Table 1**). These results suggest that this gene family primarily functioned in the cytoplasm.

**Table 1:** The physicochemical properties of TIL proteins.

Gene name	Sequence ID	Chr.	No. of A. A	Mol. weight kDa	PI	Instability index	Aliphatic index	Hydrophilia	Subcellular localization
*AtTIL*	AT5G58070.1	5	185	21306.08	5.7	45.92	67.95	-0.661	Cytoplasm.
*BrTIL1*	Brapa10T001760.1	A10	188	21493.37	6.44	41.67	67.87	-0.638	Cytoplasm
*BrTIL2*	Brapa02T001218.1	A02	203	23495.61	5.88	41.98	62.86	-0.752	Cytoplasm
*BrTIL3*	Brapa03T001168.1	A03	187	21432.23	5.97	38.69	67.75	-0.64	Cytoplasm
*BraTIL1*	Bra020391.1	A02	180	20735.38	5.6	43.8	69.28	-0.656	Extracellular
*BraTIL2*	Bra020393.1	A02	187	21561.4	6.1	41.28	66.68	-0.64	Cytoplasm
*BraTIL3*	Bra006784.1	A03	187	21432.23	5.97	38.69	67.75	-0.640	Extracellular
*BraTIL4*	Bra019518.1	A06	346	38891.66	6.54	39.74	72.69	-0.317	Cytoplasm
*BraTIL5*	Bra002674.1	A10	137	15710.04	9.25	31.72	73.21	-0.385	Extracellular
*BjTIL1*	BjuA006639	A02	262	30079.15	5.81	40.82	65.50	-0.595	Extracellular
*BjTIL2*	BjuA041941	A03	187	21432.23	5.97	38.69	67.75	-0.640	Cytoplasm
*BjTIL3*	BjuA022753	A06	346	38891.66	6.54	39.74	72.69	-0.317	Extracellular
*BjTIL4*	BjuB048072	B02	188	21488.25	6.11	39.45	67.93	-0.676	Cytoplasm
*BjTIL5*	BjuB011793	B05	187	21310.16	6.43	41.66	68.29	-0.551	Cytoplasm
*BjTIL6*	BjuB041074	B08	187	21502.38	5.97	40.03	67.75	-0.646	Cytoplasm
*BnTIL1*	BnaA02g07880D	A02	249	28406.04	4.9	43.2	68.92	-0.565	Extracellular
*BnTIL2*	BnaA02g07900D	A02	178	20512.16	5.44	38.46	66.24	-0.616	Cytoplasm
*BnTIL3*	BnaA03g09920D	A03	187	21432.23	5.97	38.69	67.75	-0.64	Cytoplasm
*BnTIL4*	BnaA06g20710D	A06	346	38963.72	6.25	41.1	72.69	-0.326	Extracellular
*BnTIL5*	BnaA10g29280D	A10	188	21466.26	6.43	40.71	66.33	-0.662	Cytoplasm
*BnTIL6*	BnaC02g10970D	C02	181	20844.52	5.29	40.66	70	-0.696	Cytoplasm
*BnTIL7*	BnaC02g10990D	C02	187	21490.32	6.1	37.3	67.22	-0.621	Cytoplasm
*BnTIL8*	BnaC09g33600D	C09	185	21109.88	6.1	41.52	67.41	-0.614	Cytoplasm

### 3.2 Phylogenetic analysis of TIL genes

**Figure 1:**
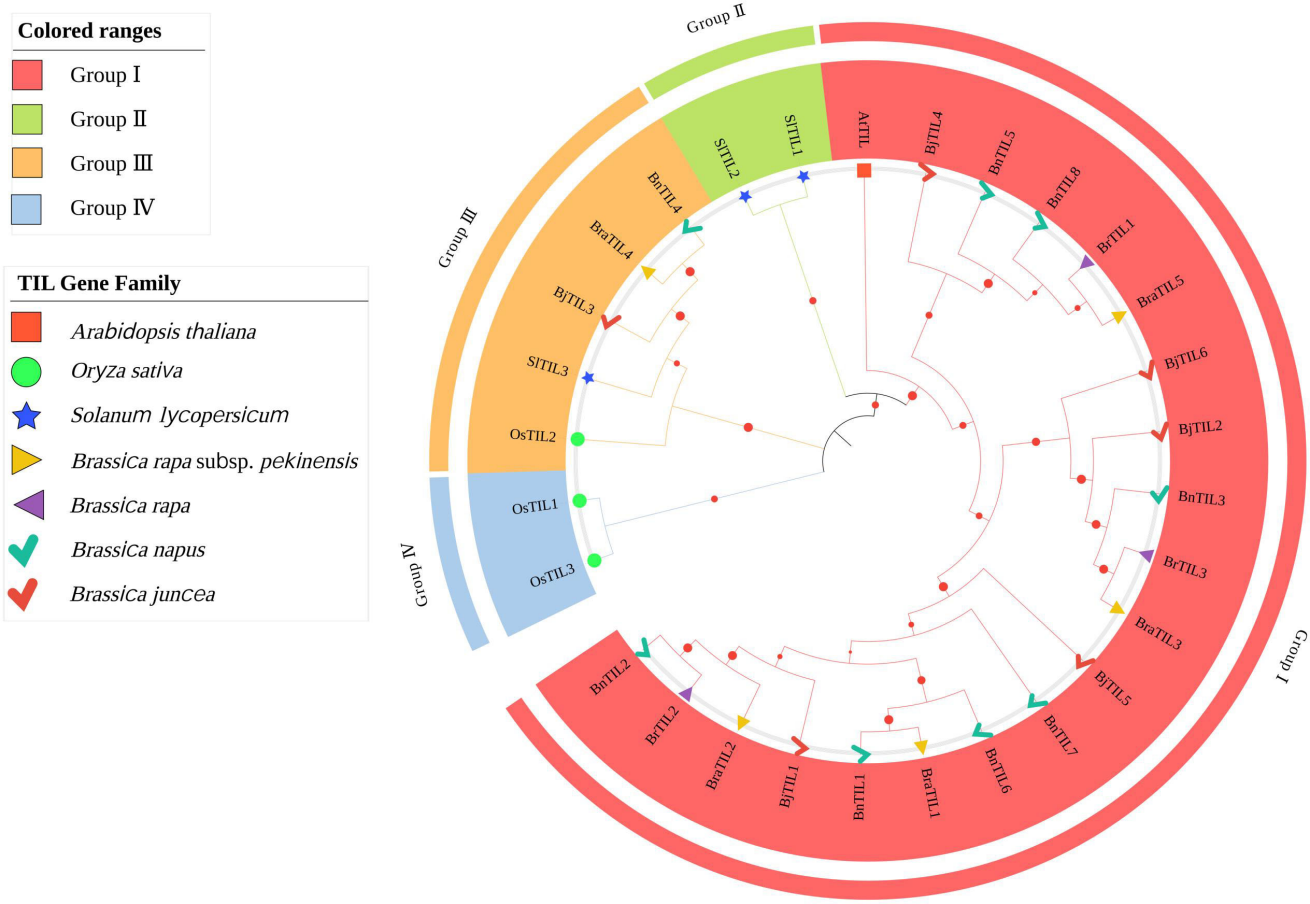
The phylogenetic analysis of TIL proteins from diverse species. Distinct clades are color-coded, and different species are represented by unique geometric shapes with specific colors.

To elucidate the evolutionary relationships of the TIL gene family, a phylogenetic tree was constructed using 23 TIL proteins from five Brassicaceae species, along with three *OsTIL* proteins from rice (*Oryza sativa*) and three *SlTIL* proteins from tomato (*Solanum lycopersicum* L.). The tree was divided into four major clades, designated I–IV (**Figure 1**, **Supplementary Table 2**). Clade I contained the most members (20), followed by Clade III (5), while Clades II and IV contained the fewest members, with 2 members each. The phylogenetic analysis revealed that Clade III contained TIL proteins from both monocots (rice) and dicots (tomato, *Brassica rapa* subsp. *pekinensis*, *Brassica juncea*, *Brassica napus*). The TIL proteins from the five Brassicaceae species (dicots) were primarily clustered in Clade I, which exhibited a greater genetic distance from Clade IV (monocots) but showed a closer phylogenetic relationship with Clade II (also dicots). These findings indicated that TIL genes existed prior to the divergence of monocots and dicots. The conserved presence of TIL genes in Clade III across both plant groups suggested that their functions were highly conserved. Furthermore, the predominant clustering of Brassicaceae TIL proteins in Clade I implied potential functional conservation within this family.

### 3.3 Analysis of gene structure and conserved motifs in TIL genes

**Figure 2:**
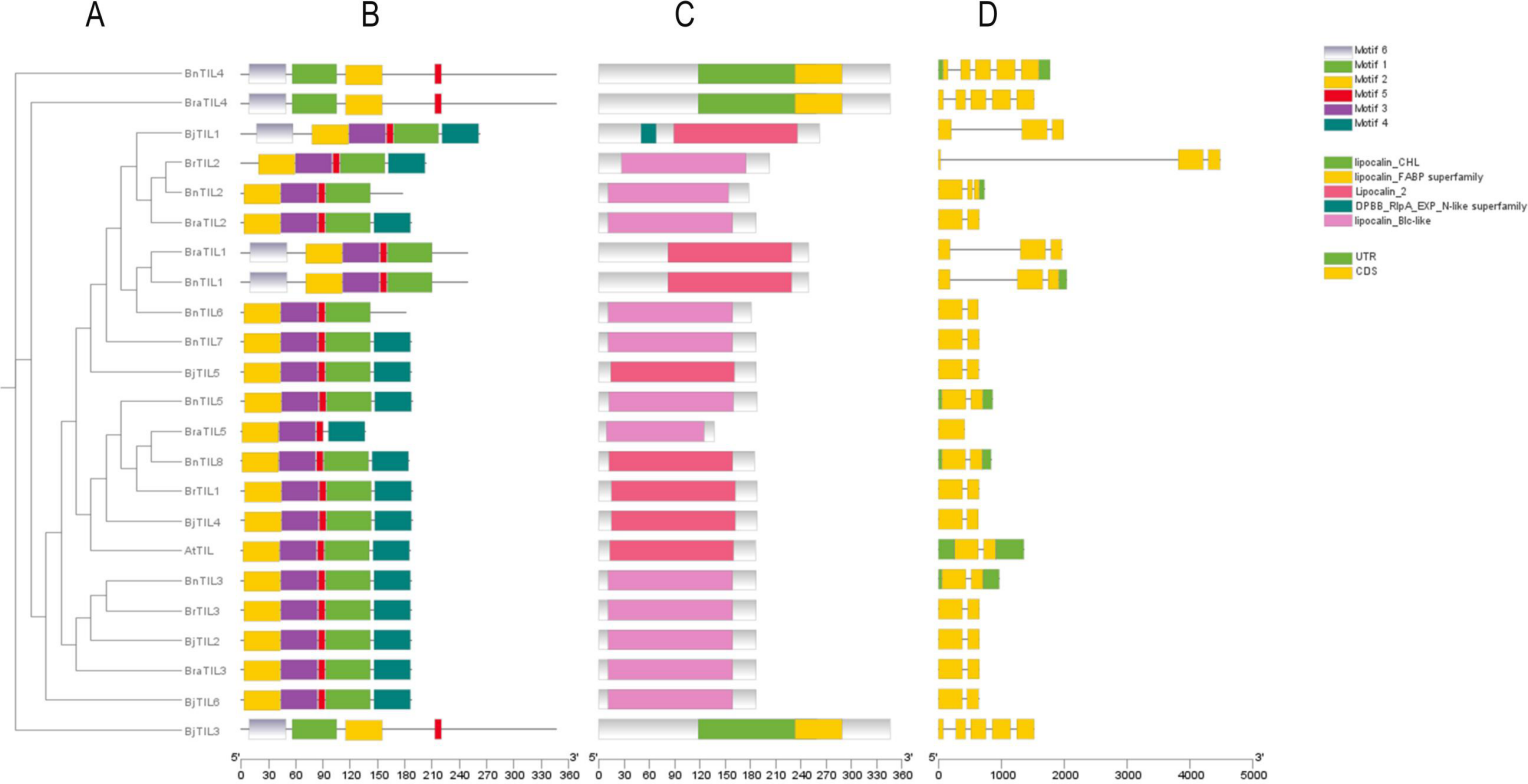
Gene structure, conserved domains, and conserved motifs of TIL genes **(A)** Phylogenetic tree of TIL genes. **(B)** Conserved motifs of TIL proteins. **(C)** Domains of TIL proteins. **(D)** Gene structure of TIL genes.

Based on phylogenetic relationships, gene structure and conserved motif analyses were conducted for the 23 TIL genes from five Brassicaceae species. The phylogenetic tree demonstrated close homology among the TIL proteins (**Figure 2A**). Conserved motif analysis revealed that all TIL members contained Motif 1, Motif 2, and Motif 5. However, certain motifs were exclusive to specific clades; for instance, Motif 3 was only identified in Clade I (**Figure 2B**). Further analysis showed that the Clade III members (*BraTIL4*, *BjTIL3*, and *BnTIL4*) possessed the lipocalin_FABP superfamily and lipocalin_CHL conserved domains. In contrast, the remaining 20 TIL proteins contained either Lipocalin-2 or lipocalin_Blc-like conserved domains (**Figure 2C**), suggesting potential functional divergence between these clades. Gene structure analysis indicated that Clade I members contained fewer introns (0–2), while Clade III members possessed a higher number (4) (**Figure 2D**), reflecting increased structural diversity that arose during evolutionary divergence. These results were consistent with the phylogenetic classification and suggested that TIL proteins within the same clade performed similar biological functions.

### 3.4 Analysis of cis-acting elements in TIL genes

**Figure 3:**
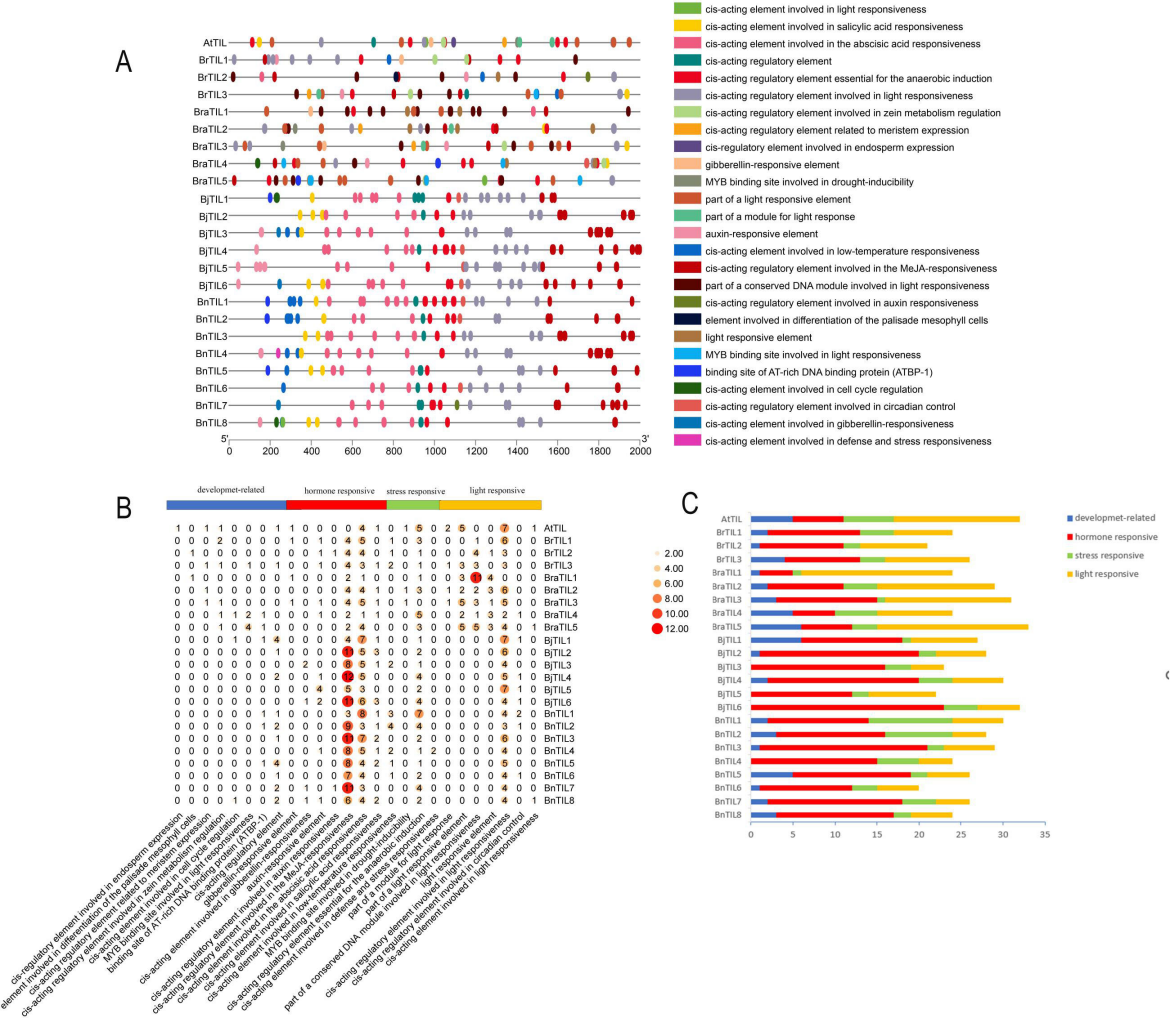
Analysis of cis-acting elements in TIL gene promoters. **(A)** Types of cis-acting elements in TIL genes. **(B)** Statistical distribution of cis-acting element types in TIL genes. **(C)** Analysis of four types of promoter *cis*-acting elements in TIL genes.

A comprehensive analysis of *cis*-acting elements was conducted in the promoter regions (2,000 bp upstream of the transcription start site) of all 23 TIL genes to investigate their potential functions. The analysis identified 26 distinct types of response elements (**Figure 3A**, **Supplementary Table 3**), which were categorized into four main groups: light-responsive, hormone-responsive, stress-responsive, and development-related elements. This finding indicated that the expression of TIL gene family members is coordinately regulated by multiple *cis*-acting elements. Further analysis revealed that light-responsive elements, ABA-responsive elements, and MeJA-responsive elements were widely distributed across all 23 genes. Notably, all three TIL genes from *Brassica rapa* contained low-temperature responsive elements, suggesting a potential association with cold acclimation during species evolution. This discovery indicated that TIL genes might contribute to this species’ capacity for low-temperature tolerance (**Figure 3B**). Furthermore, every TIL gene was found to contain at least one stress-responsive element (**Figure 3C**). Collectively, these results demonstrated that the expression of TIL genes is regulated by a diverse set of *cis*-acting elements, underscoring the extensive involvement of this gene family in plant responses to environmental stresses and hormonal signals.

### 3.5 Chromosomal distribution and evolutionary analysis of TIL genes in Brassicaceae

**Figure 4:**
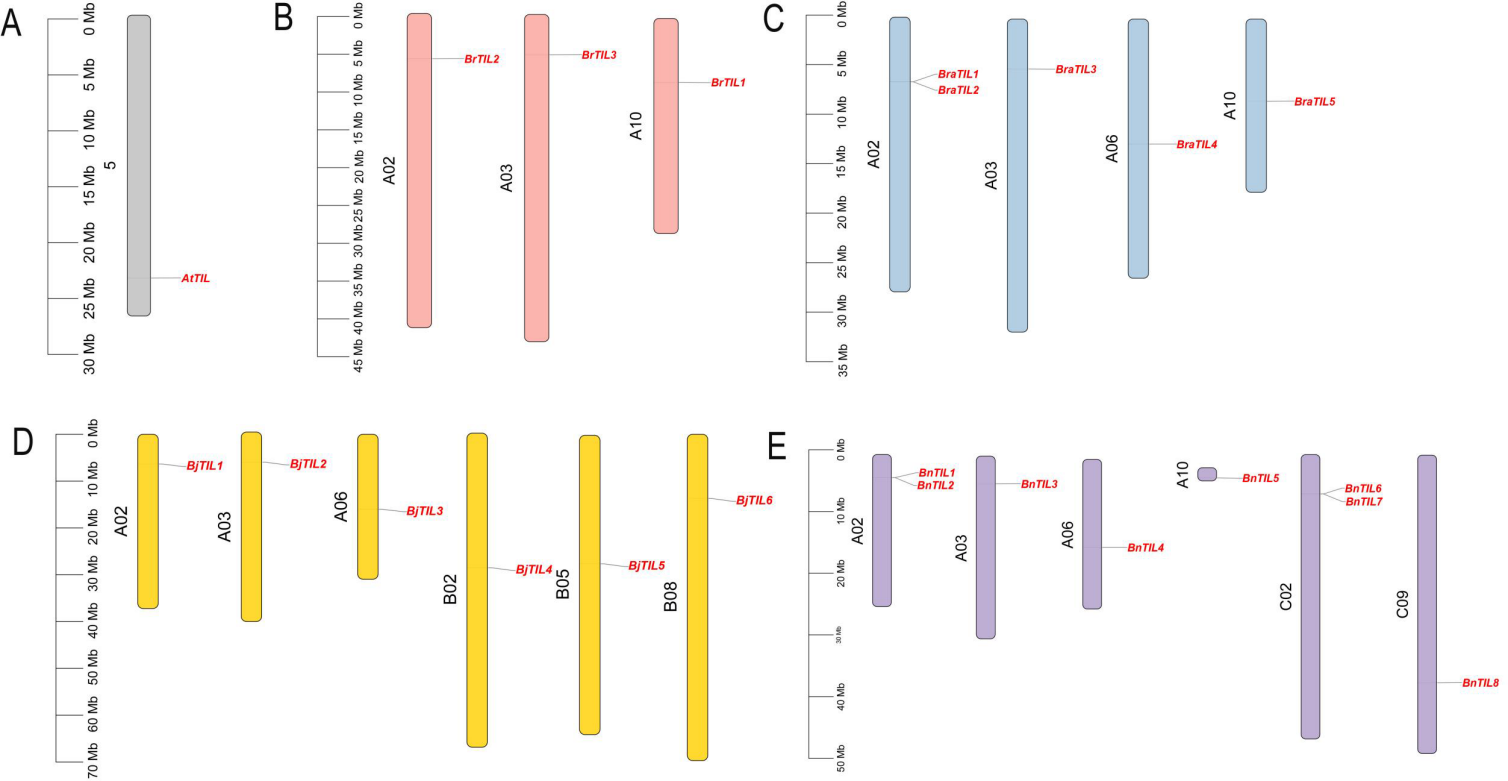
Chromosomal distribution of TIL gene family members in five Brassicaceae species. **(A)** Chromosomal localization of *AtTIL* genes in *Arabidopsis thaliana*. **(B)** Chromosomal localization of BrTIL genes in *Brassica rapa***(C)** Chromosomal localization of BraTIL genes in *Brassica rapa* subsp*. pekinensis*. **(D)** Chromosomal localization of BjTIL genes in *Brassica juncea*. **(E)** Chromosomal localization of BnTIL genes in *Brassica napus*.

The chromosomal distribution of TIL genes was analyzed across five Brassicaceae species. *Arabidopsis thaliana* contained a single *AtTIL* gene on chromosome 5 (**Figure 4A**), *Brassica rapa* harbored three *BrTIL* genes on chromosomes A02, A03, and A10 (**Figure 4B**), while its subspecies, *Brassica rapa* subsp. *pekinensis*, possessed five *BraTIL* genes on chromosomes A02, A03, A06, and A10 (**Figure 4C**). In *Brassica juncea*, six *BjTIL* genes were located on chromosomes A02, A03, A06, B02, B05, and B08 (**Figure 4D**), and *Brassica napus* contained eight *BnTIL* genes distributed across chromosomes A02, A03, A06, A10, C02, and C09 (**Figure 4E**). To understand the mechanisms of gene family expansion, duplication events were investigated. The analysis identified one tandem-duplicated gene pair in *Brassica rapa* subsp. *pekinensis* and two in *Brassica napus* (**Supplementary Table 4**). No segmental duplications were detected, indicating that tandem duplication was a major evolutionary force driving the expansion of the TIL gene family in these species.

**Figure 5:**
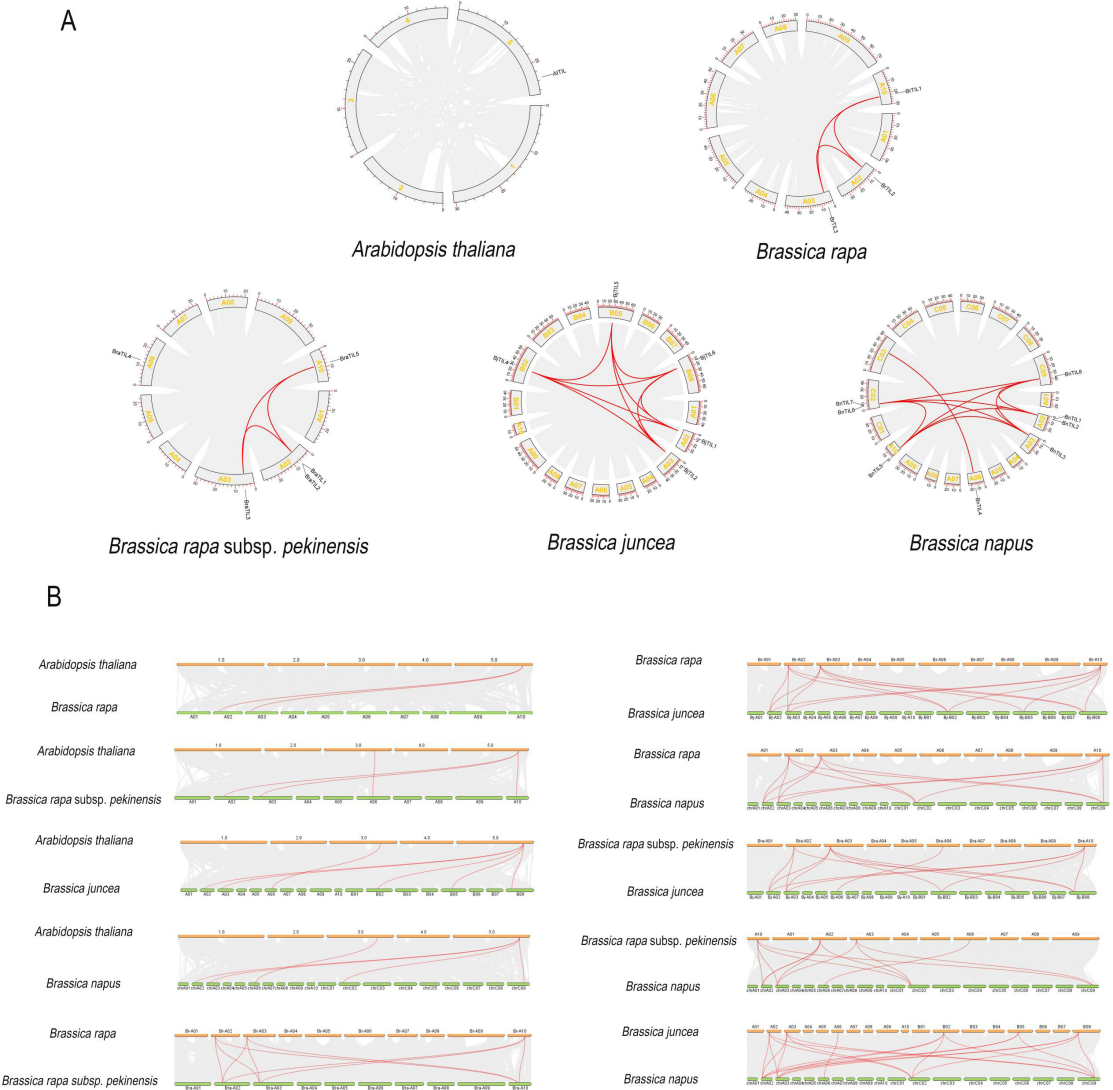
The collinearity analysis of TIL genes. **(A)** Intraspecific collinearity. **(B)** Interspecific collinearity.

Further intra-genomic collinearity analysis of TIL genesidentified 3, 3, 10, and 11 collinear gene pairs in *Brassica rapa*, *Brassica rapa* subsp. *pekinensis*, *Brassica junce*a, and *Brassica napus*, respectively, while no such events were detected in *Arabidopsis thaliana* (**Figure 5A**, **Supplementary Table 5**). These widely distributed collinear pairs further contributed to the family’s expansion. An examination of Ka/Ks ratios for these collinear pairs revealed that all values were less than 1 (**Supplementary Table 6**), indicating that the TIL genes had undergone purifying selection and possessed evolutionarily conserved functions. Interspecific collinearity analysis revealed the fewest collinear gene pairs (2) between *Arabidopsis thaliana* and *Brassica rapa*, and the most (20) between *Brassica junce*a and *Brassica napus* (**Figure 5B**, **Supplementary Table 7**), suggesting that many of these genes descended from shared ancestral genes. Furthermore, the number of collinear gene pairs increased from that in *Arabidopsis thaliana* to those in the more complex Brassica species, reflecting the accumulation of genomic duplication events during evolution. Given that *AtTIL1* in *Arabidopsis thaliana* is known to confer abiotic stress tolerance (Charron et al., 2008). TIL genes in *Brassica rapa, Brassica rapa* subsp. *pekinensi*, *Brassica junce*a, and *Brassica napus* likely perform similar, conserved roles in abiotic stress tolerance.

### 3.6 Expression patterns of TIL genes in Brassicaceae

**Figure 6:**
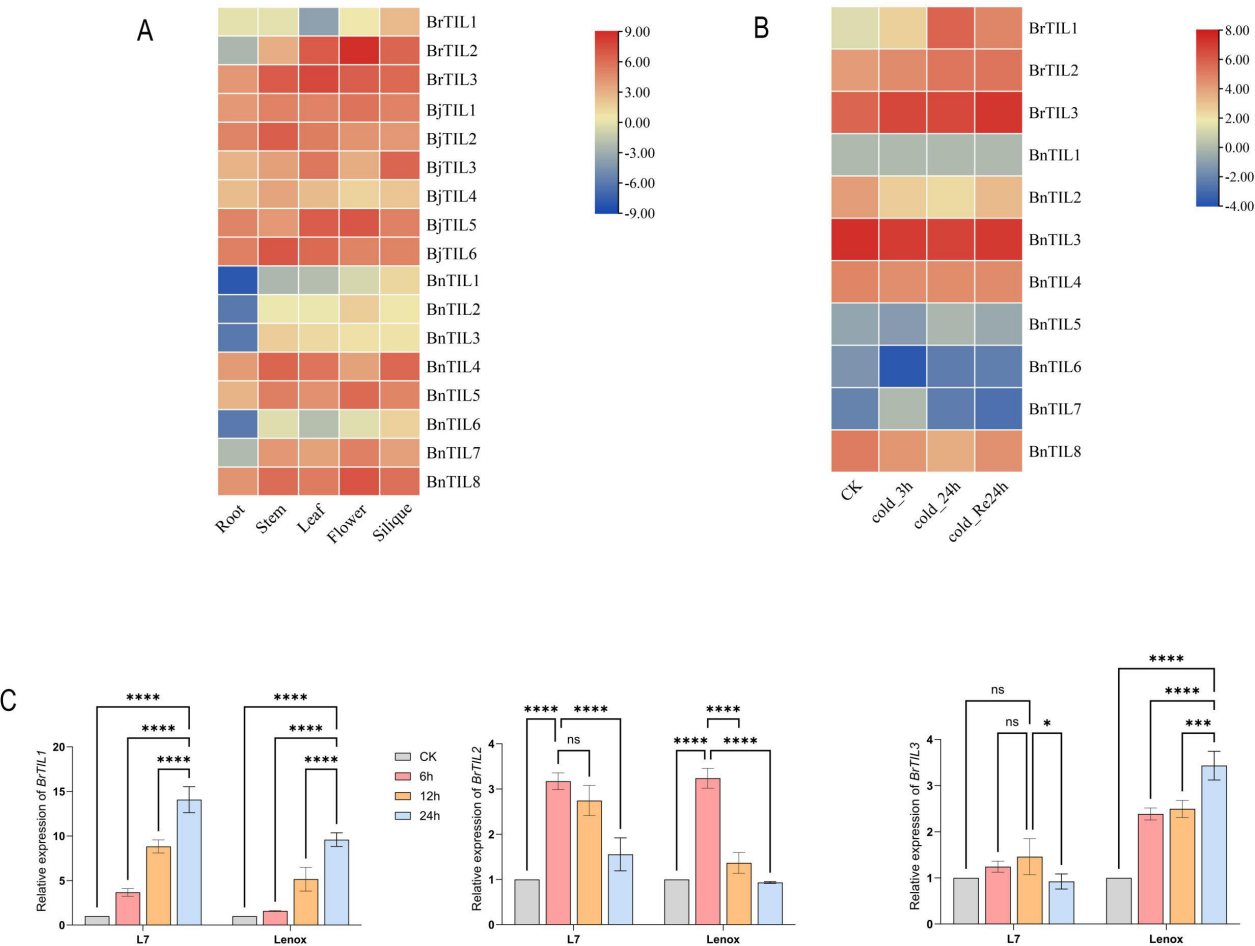
Gene expression status of TIL. **(A)** Detection of TIL gene expression in different tissues throughout. **(B)** Levels of TIL gene expression in *Brassica rapa* and *Brassica napus* subjected to cold stress. **(C)** Expression levels of BrTIL genes in *Brassica rapa* facing cold stress. Data in the figure represents the average of three independent replicates. Error bars indicate the standard deviation of the three replicates. Statistical significance between treatments was indicated by different symbols: ns indicates *p* > 0.05, no significant difference; *, **, ***, and **** indicate significant difference at the levels of *p* < 0.05, p < 0.01, p < 0.001, and *p* < 0.0001, respectively.

To further investigate the TIL gene family, we analyzed the expression patterns of 17 TIL genes across various tissues of *Brassica rapa*, *Brassica juncea*, and *Brassica napus*. The results revealed distinct tissue-specific expression profiles. In *Brassica rapa*, *BrTIL2* was highly expressed in flowers, while *BrTIL3* showed high expression in leaves. In *Brassica juncea*, although all six *BjTIL* genes were expressed at high levels, *BjTIL5* and *BjTIL6* exhibited the highest expression in flowers and stems, respectively. In *Brassica napus*, *BnTIL8* was highly expressed in flowers, whereas the expression of *BnTIL1*, *BnTIL2*, *BnTIL3*, and *BnTIL6* was down-regulated in roots (**Figure 6A**, **Supplementary Table 8**). These tissue-specific patterns suggest that TIL genes may be functionally specialized for roles in development and environmental adaptation. Analysis of transcriptome data under cold stress showed that the expression of *BrTIL1*, *BrTIL2*, and *BrTIL3* in *Brassica rapa* leaves is induced by low temperature (**Figure 6B**, **Supplementary Table 9**). To validate these findings, we performed RT-qPCR on two *Brassica rapa* cultivars with contrasting cold tolerance: the cold-tolerant ‘Longyou 7’ and the cold-sensitive ‘Lenox’. The results demonstrated that *BrTIL1* expression was continuously up-regulated with prolonged cold treatment (0 °C), peaking at 24 hours in both cultivars (**Figure 6C**). This confirmed that *BrTIL1* is a cold-responsive gene, with its expression directly modulated by the duration of low-temperature stress.

### 3.7 Analysis of the BrTIL1 protein interaction network

**Figure 7:**
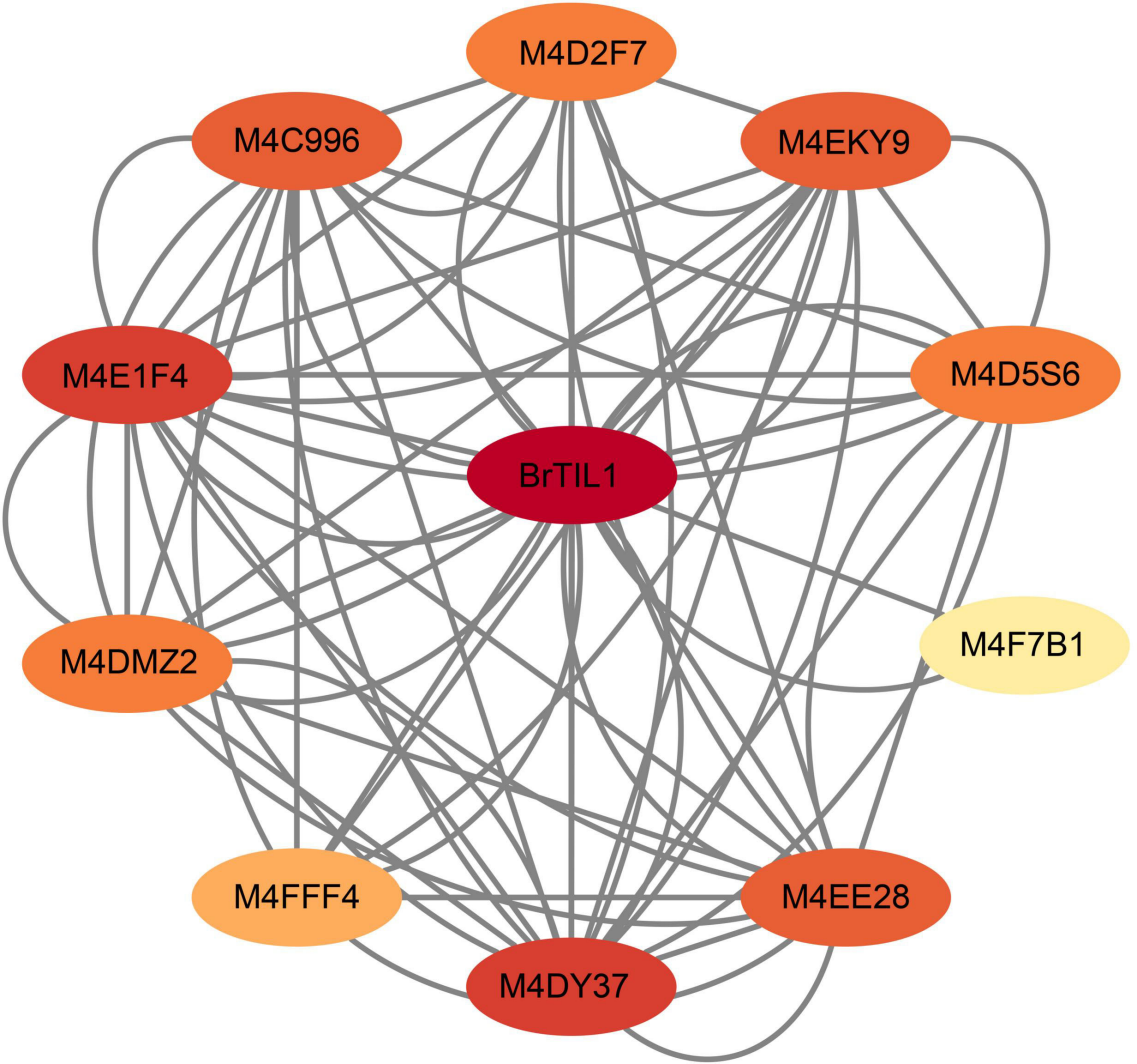
The BrTIL1 protein interaction network. Nodes represent proteins, and lines indicate interactions between them, the Confidence threshold of 0.4.

A protein-protein interaction network for BrTIL1 was predicted using the STRING database with *Brassica rapa* as the reference species. The analysis revealed that BrTIL1 potentially interacts with 10 proteins, including three transcription factors containing AP2 domains (M4D2F7, M4D5S6, and M4DMZ2), (**Figure 7**). Studies indicate that AP2 domain transcription factors serve as key regulators of low-temperature stress in *Brassica rapa* (Jaglo et al., 2001; Song et al., 2016; Mooney et al., 2019). Therefore, we hypothesize that BrTIL1 may participate in low-temperature stress signaling mechanisms by binding to AP2 domain transcription factors.

### 3.8 Overexpression of *BrTIL1* promotes root-to-shoot ratio in transgenic *Arabidopsis thaliana*

To investigate the biological function of *BrTIL1*, we generated homozygous *BrTIL1*-overexpressing *Arabidopsis thaliana* lines through Agrobacterium-mediated floral dip transformation (**Supplementary Figures 1A–C**). PCR analysis confirmed the successful integration of the transgene, indicating the presence of an approximately 400 bp target band in the transgenic lines (**Supplementary Figure 1D**). Phenotypic characterization revealed that while *BrTIL1*-overexpressing lines and wild-type plants were similar at the seedling stage, the transgenic lines exhibited significant developmental alterations post-floral transition. These changes included a dwarf stature and delayed flowering and fruiting times. A notable phenotypic difference was a significantly higher root-to-shoot ratio in the *BrTIL1-*overexpression lines compared to wild-type controls (**Supplementary Figure 1E**). These preliminary results suggest that *BrTIL1* may enhance cold tolerance in transgenic *Arabidopsis thaliana* by modulating plant architecture and resource allocation, favoring root development.

### 3.9 Overexpression of *BrTIL1* enhances cold stress tolerance in *Arabidopsis thaliana*

**Figure 8:**
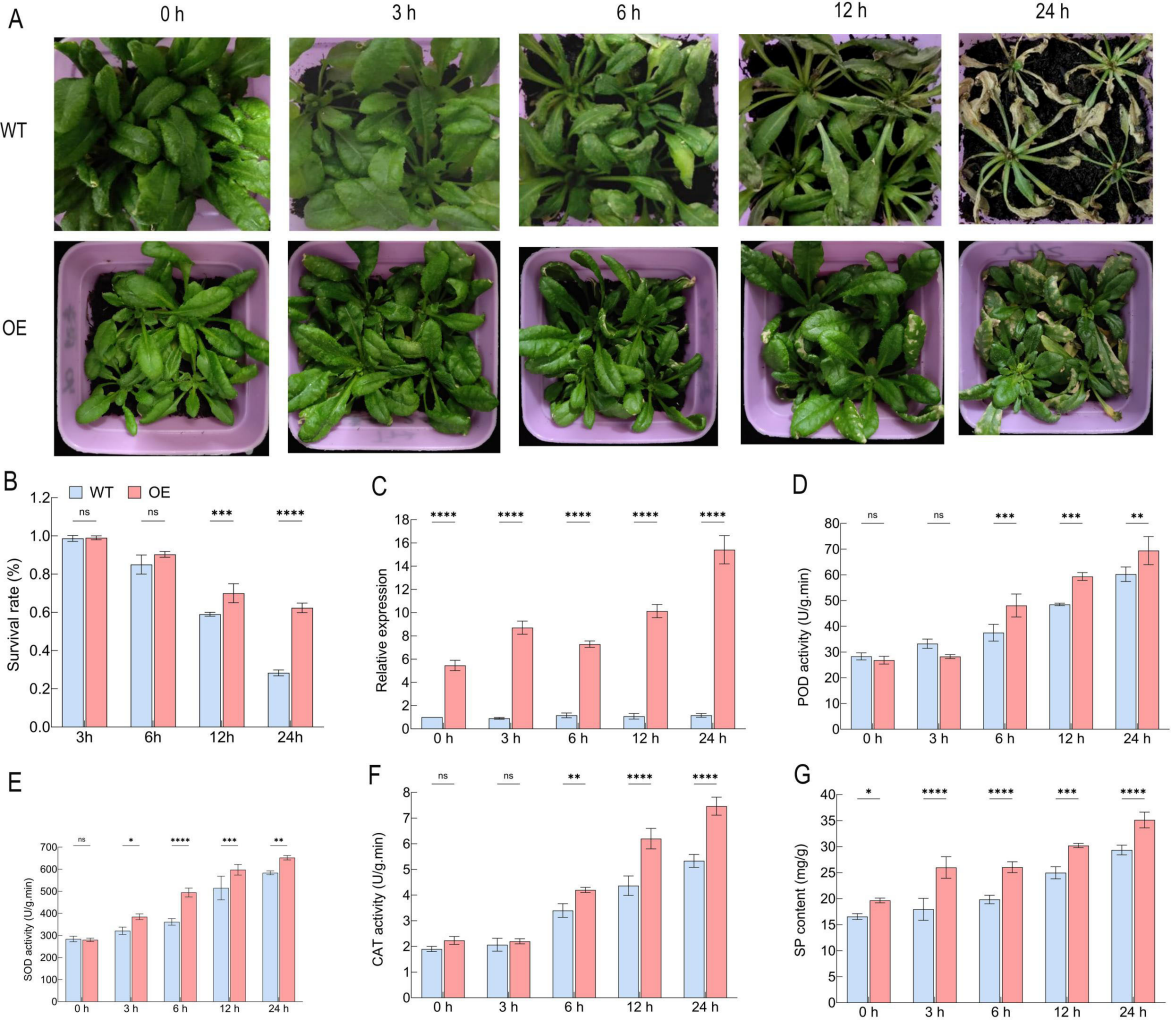
Phenotype, gene expression, and physiological activity of *BrTIL1* transgenic *Arabidopsis thaliana* after cold treatment. **(A)** Phenotype of *BrTIL1* transgenic *Arabidopsis thaliana* after cold treatment. **(B)** Survival rate of *Arabidopsis thaliana* plants after cold treatment. **(C)** Quantitative RT-PCR analysis of *BrTIL1* transgenic *Arabidopsis thaliana* under cold stress. **(D)** POD activity. **(E)** SOD activity. **(F)** CAT activity. **(G)** soluble protein content. Each sample should undergo at least three independent experiments, Data in the figure represents the average of three independent replicates. Error bars indicate the standard deviation of the three replicates. Statistical significance between treatments was indicated by different symbols: ns indicates *p* > 0.05, no significant difference; *, **, ***, and **** indicate significant difference at the levels of *p* < 0.05, p < 0.01, p < 0.001, and *p* < 0.0001, respectively.

Based on the above analysis, we selected *BrTIL1* for functional validation. To investigate the function of the *BrTIL1* gene under low-temperature stress, we subjected wild-type (WT) and *BrTIL1*-overexpressing *Arabidopsis thaliana* lines (OE) to -4 °C cold stress treatment at the seedling stage. Survival rates were recorded after one week of recovery at room temperature. The results showed no significant phenotypic differences between the two groups after 3 hours of cold treatment. However, after 24 hours, the leaves of WT plants exhibited severe yellowing and wilting, resulting in a low survival rate of only 27%. In contrast, OE lines maintained significantly better leaf integrity and exhibited a survival rate of 65% (**Figures 8A, B**), demonstrating that *BrTIL1* overexpression markedly enhances freezing tolerance. Consistent with its role in cold adaptation, *BrTIL1* expression in OE lines was strongly induced by low temperature, reaching an expression level 12.3-fold higher than in WT plants after 24 hours of cold stress treatment (**Figure 8C**). Physiological analysis revealed that antioxidant enzyme activities (SOD, POD, CAT) and soluble protein content were similar between WT and OE lines under normal conditions. However, upon cold stress, these parameters increased significantly in the OE lines. After 24 hours at -4 °C, the OE lines displayed peak POD, SOD, and CAT activities, which were 11.75%, 15.21%, and 40.00% higher than those in WT plants, respectively, alongside a 19.65% higher soluble protein content (**Figures 8D–G**). These results indicate that *BrTIL1* is a cold-induced gene that positively regulates freezing tolerance. Its overexpression enhances the plant’s capacity to scavenge ROS by boosting key antioxidant enzyme activities and osmolyte accumulation, thereby mitigating cold-induced oxidative damage.

### 3.10 Subcellular localization of BrTIL1

**Figure 9:**
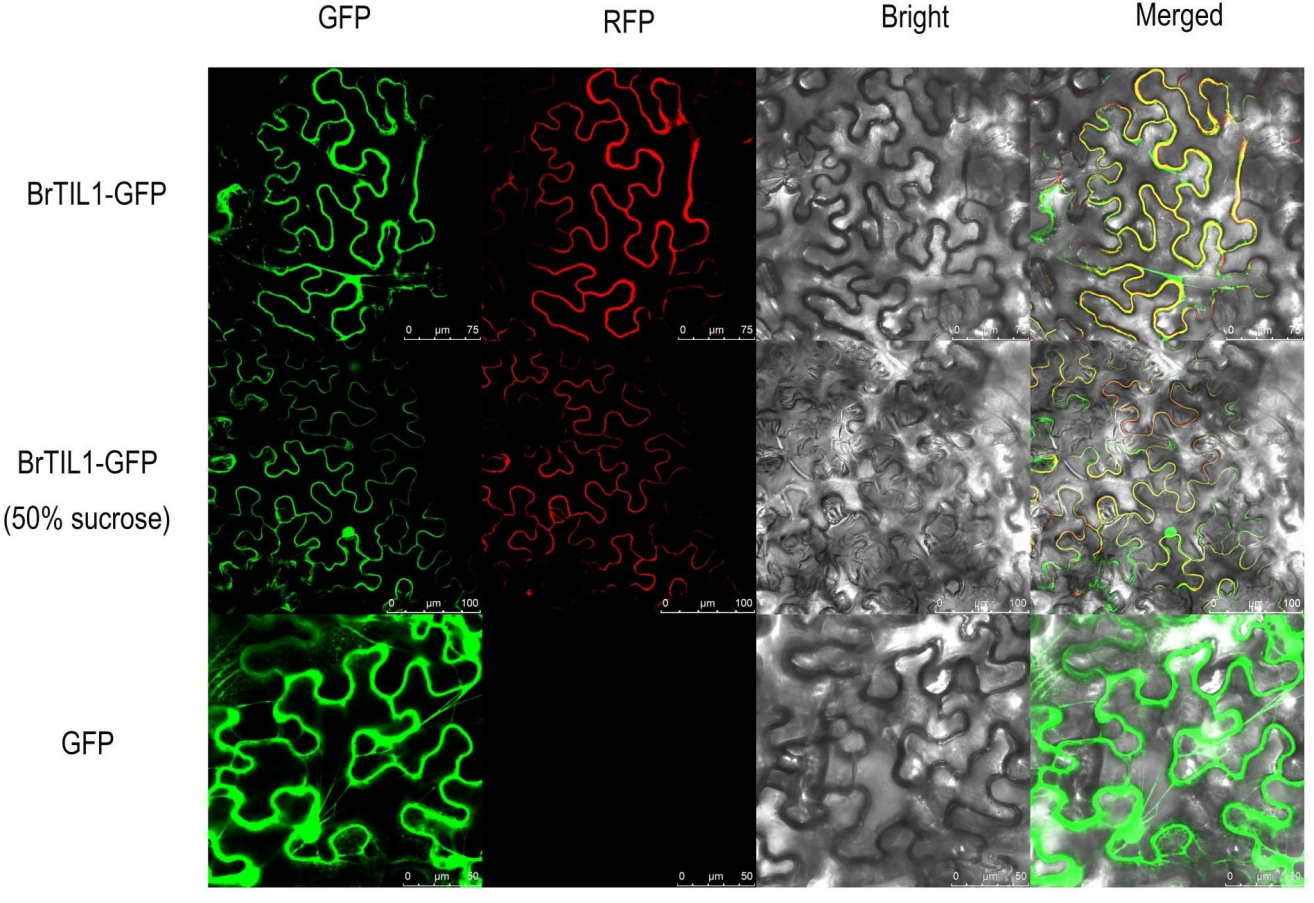
Subcellular localization of BrTIL. BrTIL1-GFP: BrTIL1-GFP fusion protein observed in normally treated tobacco leaves, scale bar 75 μm. BrTIL1-GFP (50% sucrose): BrTIL1-GFP fusion protein observed in tobacco leaves treated with 50% sucrose solution, scale bar 100 μm. GFP: GFP protein observed in normally treated tobacco leaves, scale bar 50 μm.

To determine the subcellular localization of *BrTIL1*, a pSuper1300-BrTIL1-GFP fusion construct was transiently expressed in *Nicotiana benthamiana* leaves. Confocal laser scanning microscopy showed that the green fluorescence signal of the BrTIL1-GFP fusion protein co-localized precisely with the red fluorescence of the plasma membrane marker. To confirm this localization, plasmolysis was induced by treating the leaf tissue with a 50% sucrose solution. The resulting separation of the plasma membrane from the cell wall demonstrated that the BrTIL1-GFP signal remained exclusively associated with the membrane (**Figure 9**). These results conclusively identifyed BrTIL1 as a plasma membrane-localized protein, supporting a potential role in membrane protection and stabilization, particularly under stress conditions.

### 3.11 Screening and identification of BrTIL1-interacting proteins

**Figure 10:**
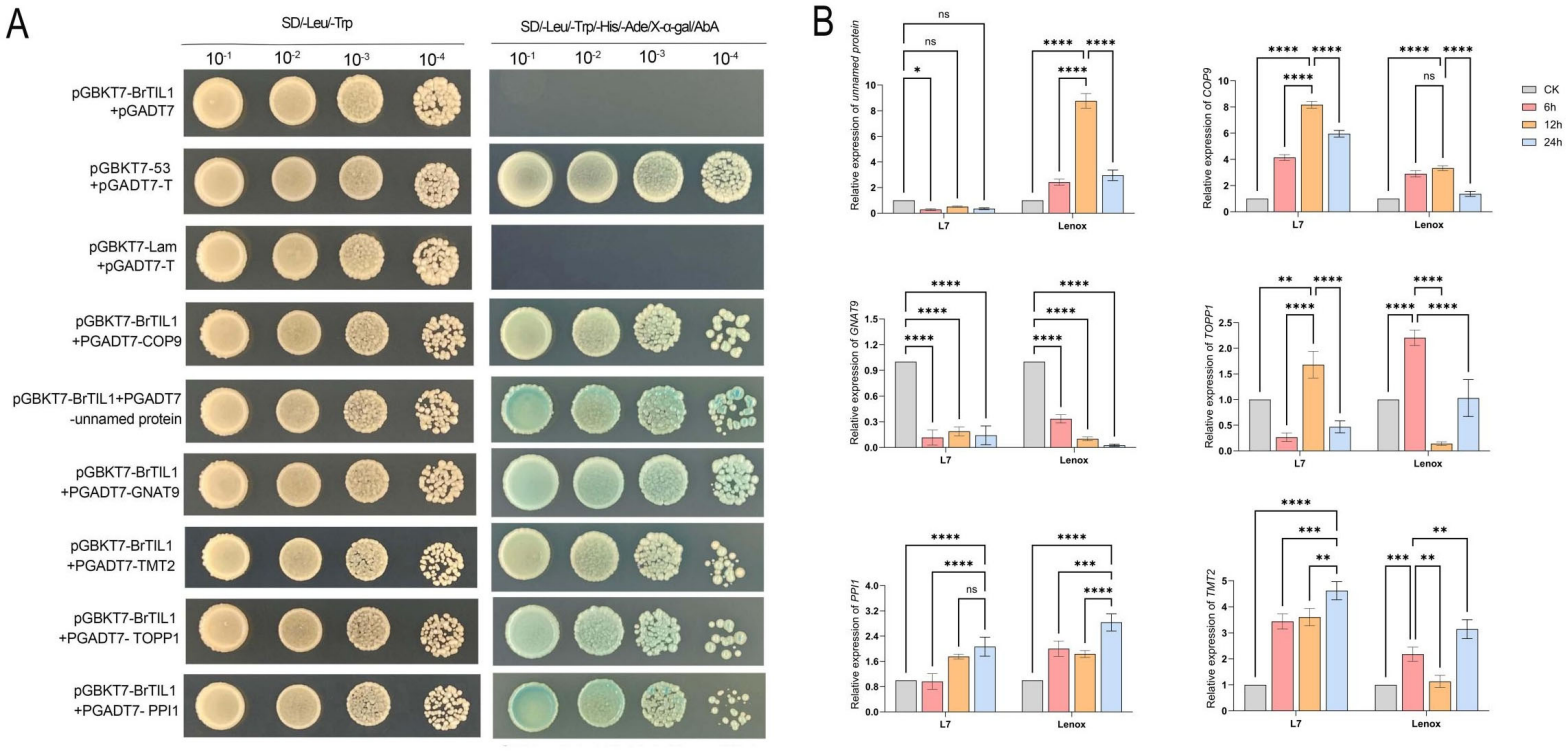
Screening and identification of BrTIL1-interacting proteins. **(A)** One-to-one verification of BrTIL1 positive clones by yeast two-hybrid assay. **(B)** Expression of *BrTIL1*-interacting protein genes under low temperature. Expression of *BrTIL1*-interacting protein genes under low temperature. Data in the figure represents the average of three independent replicates. Error bars indicate the standard deviation of the three replicates. Statistical significance between treatments was indicated by different symbols: ns indicates *p* > 0.05, no significant difference; *, **, ***, and **** indicate significant difference at the levels of *p* < 0.05, p < 0.01, p < 0.001, and *p* < 0.0001, respectively.

To identify proteins that interact with BrTIL1 in *Brassica rapa*, a yeast two-hybrid (Y2H) cDNA library was constructed. Through self-activation validation (**Supplementary Figure 2A**), re-screening (**Supplementary Figure 2B**), and GO annotation with KEGG functional analysis (**Supplementary Figures 2C, D**), combined with molecular feature and signaling pathway analysis, six candidate proteins were selected for yeast single-hybrid validation with pGBKT7-BrTIL1. The results indicated that the BrTIL1 protein specifically interacted wit COP9, unnamed protein, the GNAT9, TMT2, TOPP1, and PPI1 in yeast cells (**Figure 10A**). Gene expression regulation is a fundamental component of the plant stress response. To investigate the relationship between *BrTIL1* and its interacting partners, we analyzed the expression patterns of the six confirmed interacting protein genes under cold stress using RT-qPCR. The results revealed distinct expression profiles among the interactors (**Figure 10B**). Notably, the expression of *COP9* and *TMT2* closely mirrored that of *BrTIL1*, both showing significant upregulation in response to cold stress. This induction was more pronounced in the cold-tolerant cultivar ‘Longyou 7’ than in the cold-sensitive ‘Lenox’. Specifically, *COP9* expression peaked at 12 hours, with 8.2-fold and 3.3-fold increases in ‘Longyou 7’ and ‘Lenox’, respectively. Similarly, TMT2 expression peaked at 24 hours, with 4.6-fold and 3.1-fold upregulation in the respective cultivars. In contrast, other interactors displayed divergent expression. The unnamed protein gene was downregulated in ‘Longyou 7’ but upregulated in ‘Lenox’. PPI1 was upregulated in both cultivars but showed overall higher expression in ‘Lenox’, potentially indicating a role in cold stress perception rather than tolerance. Meanwhile, GNAT9 expression was significantly suppressed, and TOPP1 exhibited fluctuating expression levels under cold stress. Collectively, these findings demonstrate that BrTIL1-interacting proteins display diverse and cultivar-specific expression patterns under low-temperature stress, implicating them in a complex regulatory network governing cold adaptation.

## 4 Discussion

TIL genes are well-established as key factors in plant responses to abiotic stress, such as in *Arabidopsis thaliana*, *Chrysanthemum × morifolium*, and *Triticum aestivum* (Charron et al., 2005; Chi et al., 2009; Huang et al., 2021). However, reports on TIL genes in *Brassica rapa* are scarce, and studies on the related TIL gene family remain limited. This study addresses this gap by performing a systematic genomic and functional analysis of the TIL gene family in five Brassicaceae species, with a focus on the *Brassica rapa BrTIL1* gene. The findings revealed that TIL family expansion and diversification in Brassicaceae primarily occurred through polyploidy events. Strong evidence indicated that *BrTIL1* functions as a core regulator of cold tolerance, potentially by stabilizing cellular membrane structures and coordinating complex protein interaction networks. This study not only elucidated the evolutionary dynamics of the TIL family but also identified *BrTIL1* as a valuable genetic resource for the molecular breeding of cold-tolerant *Brassica rapa*.

The variation in TIL gene copy number across the five species from a single gene in *Arabidopsis thaliana* to eight in the allotetraploid *Brassica napus* correlates directly with their genomic complexity. This pattern is a hallmark of the whole-genome triplication (WGT) event experienced by the ancestral Brassica species, which provided the raw genetic material for subsequent diversification (Town et al., 2006). The phylogenetic relationships among the identified TIL genes further reflect the well-documented hybridization history of the genus. For instance, the orthologous relationship between *BraTIL4* (AA genome), *BjTIL3* (AABB genome), and *BnTIL4* (AACC genome) strongly suggests that this specific TIL lineage originated in the AA sub-genome and was conserved through the hybridization events that formed *Brassica juncea* and *Brassica napus* (Liu et al., 2021; Zhang et al., 2024). Both apples and pears underwent whole-genome duplication (WGD) events during their evolutionary history, resulting in a doubling of their chromosome number and an increase in bHLH genes, consistent with our findings (Mao et al., 2017; Zhang et al., 2018; Wu et al., 2022). This conservation underscores the potential functional importance of this particular clade and illustrates how polyploidization has served as a foundation for adaptive evolution in Brassica species.

Research has revealed that tandem duplication and segmental duplication may be the primary drivers of gene family expansion and novel gene generation (Hao and He, 2024). For instance, in cherries, the ChbHLH gene family expanded its membership through tandem and segmental duplication. This phenomenon was similarly observed in the present study, with evidence visible in the duplicate gene pairs of *Brassica rapa* subsp. *pekinensis* and *Brassica napus* (Rizwan et al., 2022; Hao and He, 2024). All collinear TIL genes underwent intense purifying selection (Ka/Ks < 1), indicating that their core biochemical functions are crucial and have been strictly conserved during the evolution of the Brassicaceae family. The collinearity of BrTILs with those in other species was also analyzed. Compared with *Arabidopsis thaliana* and *Brassica rapa*, *Brassica napus* shares more collinear genes with *Brassica juncea* and *Brassica rapa* subsp. *pekinensis*, consistent with the relative evolutionary relationships among these species.

We found that the *BrTIL1* gene exhibited significantly elevated expression under cold stress (**Figure 6**), demonstrating cold-inducibility similar to that of TIL genes in plants such as *Arabidopsis thaliana*, rice, and wheat (Charron et al., 2005; Huang et al., 2021). From an evolutionary perspective, the TIL gene family is closely related between *Brassica rapa* and *Arabidopsis thaliana* (Chalhoub et al., 2014). Furthermore, by predicting protein interaction networks, we discovered that the BrTIL1 protein may interact with AP2 domain-containing transcription factors (**Figure 7**). We speculate that *BrTIL1* may possess a cold resistance function. Shifting from evolutionary analysis to functional validation, we note that studies have reported that TIL1 is a key mediator of cold tolerance. Transfection of the *EuTIL1* gene into *Arabidopsis thaliana* reduces malondialdehyde (MDA) content, increases maximum soluble sugar (SS) content, alters antioxidant enzyme activity, and positively regulates cold tolerance in *Arabidopsis thaliana* (Wu and Zhao, 2023). In our study, BrTIL1 subcellular localization to the plasma membrane is consistent with its proposed role in the primary site of cold perception and injury (Abo-Ogiala et al., 2014; Ji et al., 2024). The dramatic improvement in survival and reduction in leaf damage in *BrTIL1*-overexpressing *Arabidopsis thaliana* lines under freezing stress provides direct evidence of its protective function. Physiologically, this tolerance is linked to a significantly enhanced capacity for ROS scavenging. Transgenic plants exhibited a more robust and rapid increase in the activities of key antioxidant enzymes (SOD, POD, CAT) and soluble protein content upon cold exposure (Huang et al., 2021). This suggests that *BrTIL1* bolsters the cellular antioxidant system, thereby mitigating the oxidative damage that is a major consequence of cold stress.

The novel insight from our study is the identification of a *BrTIL1*-centered protein interaction network. The six confirmed interactors connect *BrTIL1* to diverse cellular processes, positioning it as a potential signaling hub (Qin et al., 2020). Particularly compelling is the co-upregulation of *BrTIL1*, *COP9*, and *TMT2* in the cold-tolerant cultivar ‘Longyou 7’. COP9 is a component of the signalosome complex, a key regulator of ubiquitin-mediated protein degradation linked to stress and light signaling (Schulze-Niemand and Naumann, 2023; Dong et al., 2024). TMT2 is a vacuolar sugar transporter crucial for redistributing sugars for use as cryoprotectants (Wormit et al., 2007). We propose a model wherein membrane-localized *BrTIL1* perceives or responds to cold stress and, through interactions with proteins like COP9, modulates downstream signaling and stress-responsive pathways, while simultaneously influencing osmotic adjustment via TMT2. However, our work remains incomplete. The interaction between the BrTIL1 protein and the two other proteins (COP9 and TMT2) can be verified through various protein-protein interaction experiments.

## 5 Conclusion

Based on the genomic data of five cruciferous plants, a total of 23 TIL genes were identified in this study. Bioinformatics analysis revealed that the evolution of the TIL gene family in these species was closely linked to Brassica genome polyploidization, with the family exhibiting high evolutionary conservation. The *BrTIL1* gene exhibits significant upregulation under low-temperature stress. Functional studies demonstrated that *BrTIL1* overexpression enhances antioxidant enzyme activity, reduces reactive oxygen species accumulation, and significantly improves cold tolerance in transgenic plants. Furthermore, we identified six BrTIL1-interacting proteins (COP9, the unnamed protein, GNAT9, TMT2, TOPP1, and PPI1) whose encoding genes show differential expression under low-temperature stress, suggesting that they may participate in the *BrTIL1*-mediated cold response network. Collectively, this study provides both systematic insights into the TIL gene family evolution in Brassicaceae and important foundations for elucidating the molecular mechanisms underlying *BrTIL1*-mediated cold stress adaptation.

## Data Availability

The datasets presented in this study can be found in online repositories. The names of the repository/repositories and accession number(s) can be found in the article/**Supplementary Material**.
