# Supplementary figures and images for: Analysis of the TIL gene family in Brassicaceae species and functional study of BrTIL1 in cold tolerance
Source: Front Plant Sci. 2026 Feb 27;17:1794987. doi: 10.3389/fpls.2026.1794987 (PMC12982346; doi:10.3389/fpls.2026.1794987)

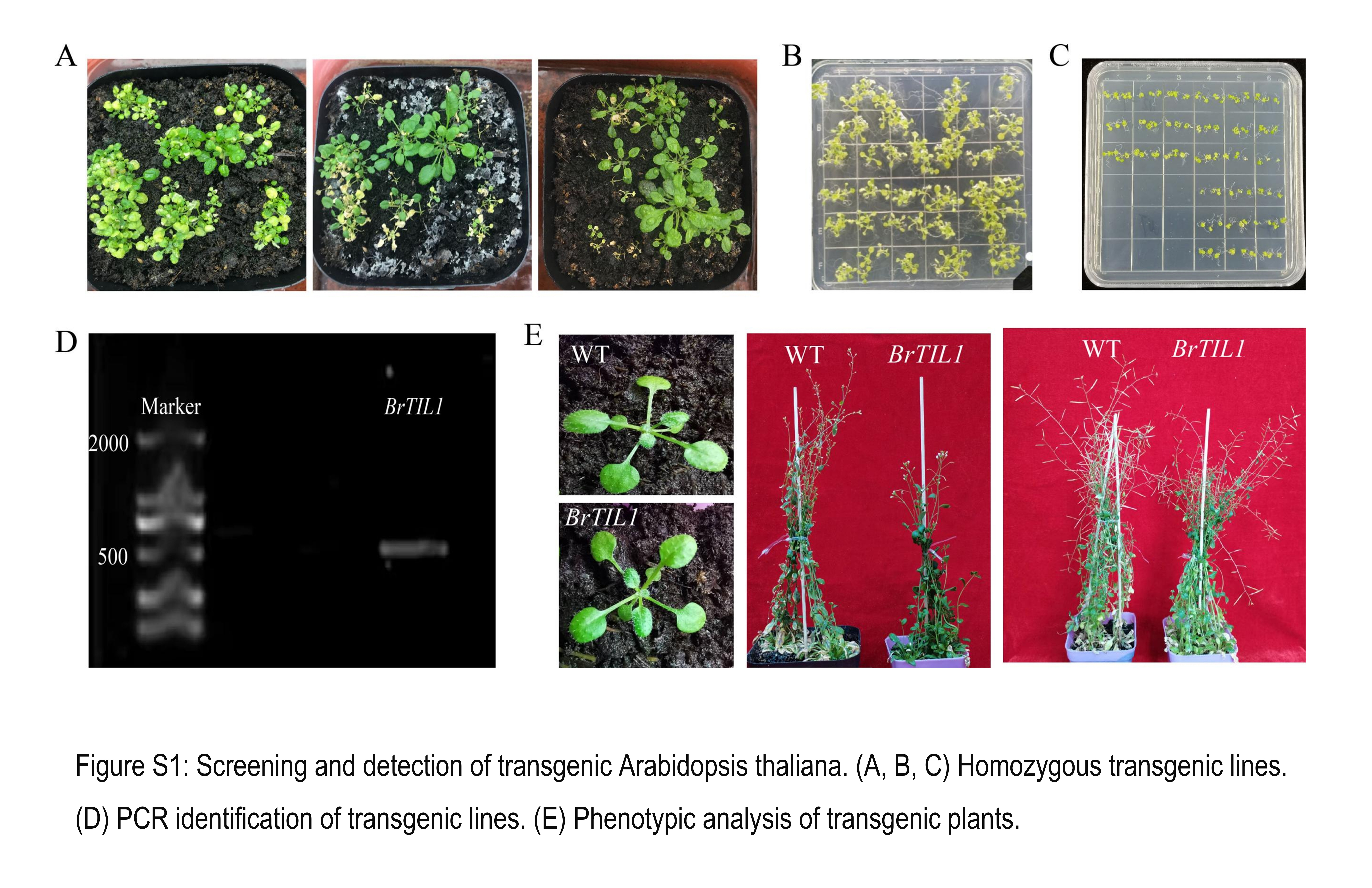

Supplement: Supplementary file 1 [file Image1.jpeg]

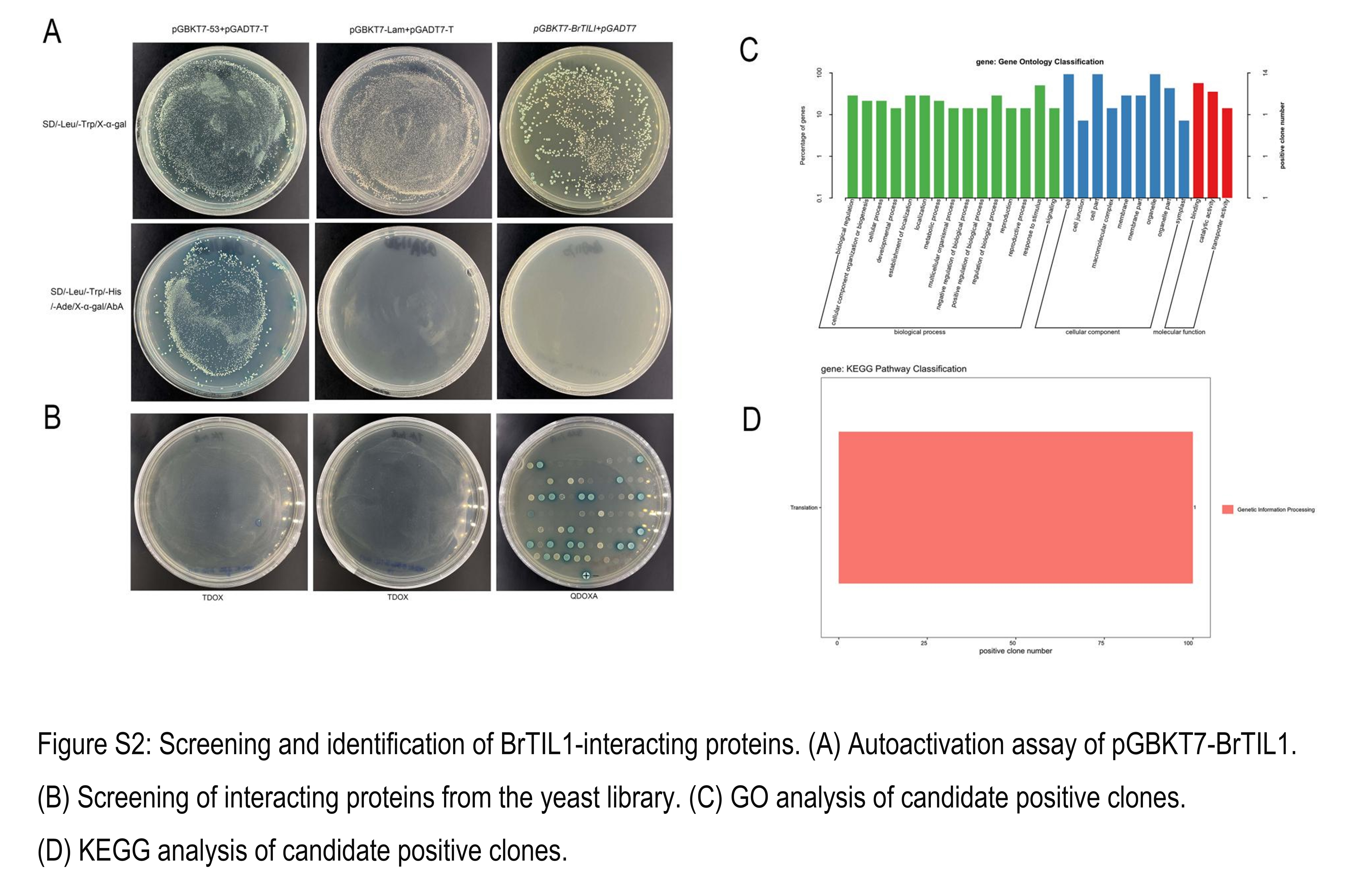

Supplement: Supplementary file 2 [file Image2.jpeg]
